# Preparation and Characterization of Novel Microgels Containing Nano-SiO_2_ and Copolymeric Hydrogel Based on Poly (Acrylamide) and Poly (Acrylic Acid): Morphological, Structural and Swelling Studies

**DOI:** 10.3390/ma15144782

**Published:** 2022-07-08

**Authors:** Tannaz Soltanolzakerin Sorkhabi, Mehrab Fallahi Samberan, Krzysztof Adam Ostrowski, Tomasz M. Majka, Marcin Piechaczek, Paulina Zajdel

**Affiliations:** 1 Department of Chemical Engineering, Ahar Branch, Islamic Azad University, Ahar P.O. Box 5451116714, Iran; tannazsoltanzakeri@gmail.com; 2 Faculty of Civil Engineering, Cracow University of Technology, 24 Warszawska Str., 31-155 Cracow, Poland; krzysztof.ostrowski.1@pk.edu.pl (K.A.O.); marcin.jaroslaw.piechaczek@gmail.com (M.P.); paulina.zajdel1@pk.edu.pl (P.Z.); 3 Department of Chemistry and Technology of Polymers, Faculty of Chemical Engineering and Technology, Cracow University of Technology, Warszawska 24, 31-155 Cracow, Poland; tomasz.majka@pk.edu.pl

**Keywords:** copolymer, hydrogel, microgel, nanocomposite, nanosilica

## Abstract

In this paper, novel microgels containing nano-SiO_2_ were prepared by in situ copolymerization using nano-SiO_2_ particles as a reinforcing agent, nanosilica functional monomer (silane-modified nano-SiO_2_) as a structure and morphology director, acrylamide (AAm) as a monomer, acrylic acid (AAc) as a comonomer, potassium persulfate (KPS) as a polymerization initiator, and N,N′-methylene bis (acrylamide) (MBA) as a crosslinker. In addition, a conventional copolymeric hydrogel based on poly (acrylamide/acrylic acid) was synthesized by solution polymerization. The microgel samples, hydrogel and nanoparticles were characterized by transmission electron microscopy (TEM), field emission scanning electron microscopy (FESEM), Fourier transform infrared (FTIR) spectroscopy, thermogravimetric analysis (TGA) and differential scanning calorimetry (DSC). A FESEM micrograph of copolymeric hydrogel showed the high porosity and 3D interconnected microstructure. Furthermore, FESEM results demonstrated that when nano-SiO_2_ particles were used in the AAm/AAc copolymerization process, the microstructure and morphology of product changed from porous hydrogel to a nanocomposite microgel with cauliflower-like morphology. According to FESEM images, the copolymerization of AAm and AAc monomers with a nanosilica functional monomer or polymerizable nanosilica particle as a seed led to a microgel with core–shell structure and morphology. These results demonstrated that the polymerizable vinyl group on nano-SiO_2_ particles have controlled the copolymerization and the product morphology. FTIR analysis showed that the copolymeric chains of polyacrylamide (PAAm) and poly (acrylic acid) (PAAc) were chemically bonded to the surfaces of the nano-SiO_2_ particles and silane-modified nano-SiO_2_. The particulate character of microgel samples and the existence of long distance among aggregations of particles led to rapid swelling and increasing of porosity and therefore increasing of degree of swelling.

## 1. Introduction

Polymeric hydrogels have a particular characteristic that make them unique materials. Hydrogels are a three-dimensional network of loosely crosslinked polymers that can absorb and retain several times even thousands of times weight of aqueous fluids due to the presence of large amounts of hydrophilic groups such as carboxyl (–COOH), sulfonic acid (–SO_3_H), amine (–NH_2_) and hydroxyl (–OH) [1,2,3]. The presence of these functional groups in the structure of hydrogels contribute to the hydrophilic character and water absorbency of hydrogels [4]. Meanwhile, microgels are hydrogel particles with their size ranging from nanometers to micrometers [5]. These particles can form colloids. Hydrogels are classified into three groups based on their composition. This group includes multipolymer interpenetrating polymeric hydrogel, copolymeric hydrogels and homopolymeric hydrogels. Hydrogels are also divided into four groups based on the network electrical charges including nonionic, ionic, amphoteric electrolyte, and zwitterion [6,7]. Hydrogels as environmental-sensitive materials can show volume transition in response to the surrounding environment (e.g., light, electric and magnetic field, temperature, pH, enzymes) [8,9,10]. These kind of revolutionary materials are widely used in environmental and separation systems (heavy metal ions remover [11]), physiological hygiene products (baby diapers and sanitary towels [12]), tissue engineering (material for scaffolds [13]), contact lenses [14], sensors and actuators [15], supercapacitors [16], slow release fertilizer [17], pharmaceuticals (controlled drug delivery [18]), agriculture and forestry (water conservation retention [19]), cosmetic industry [20] and civil engineering (sealing rod, cement [21]). During the past two decades, organic/inorganic nanocomposites have been developed to help improve the properties of conventional hydrogel and overcome their weaknesses [22]. These novel types of strong materials with excellent properties such as thermal stability and high swelling ratio are generally organic macromolecule composites with inorganic materials such as nanoparticles with nanoscale size and high surface area [23].

AAc acid and AAm are among the main monomers used in the preparation and production of commercial hydrogels. These monomers are common materials for preparing absorbent materials in industry due to their desired swelling properties. AAms as a small molecular weight are the most commonly used hydrogels [24]. Aam-based hydrogels show significant volume transition in response to external (physical and chemical) stimuli [3]. Propenoic acid or AAc monomer is crosslinked in the single or multi-component polymerization system to produce hydrogels with high water absorbing capacity. AAc has the ability of connection to the vinyl group due to its carboxylic acid group. The presence of this ionizable carboxylic acid group aids in increasing the ionic strength and sensitivity to the pH of the prepared hydrogel samples. Meanwhile, AAc monomers are used with a combination of some other polymers such as polyacrylamide to synthesize different forms of hydrogels [3,25]. In recent years, nanocomposites, which consist of polymers and nanomaterials, gained considerable attention due to synergistic effects among their components. The presence of nanomaterials in nanocomposite could result in superior properties such as high mechanical strength, good barrier properties, improved thermal stability, and so on [26]. Among the nanocomposites, polymer/nano-SiO_2_ nanocomposites have attracted much attention due to the large surface area and smooth surface of nano-SiO_2_ particles. The presence of silanol and siloxane groups on the nano-SiO_2_ surface lead to the improvement of particles’ hydrophilicity, which promote the compatibility with polymer chains. In addition, nano-SiO_2_ can function as structure and morphology directors in nanocomposite synthesis. The results of many studies have indicated that the performance of nanocomposite material could be considerably improved by combination or copolymerization with a functional monomer containing nano-SiO_2_ [27]. The nanocomposite materials containing nano-SiO_2_ such as nylon 6 [28], polyaniline [29], styrene butadiene rubber [30], polyimide [31], polyethylene terephthalate [32], may show more satisfactory thermal stability, toughness, and strength.

Nanocomposite systems can be synthesized by various synthesis routes, thanks to the ability to combine different ways to introduce each phase. The organic component can be introduced as (1) a precursor, which can be a monomer or an oligomer, (2) a preformed linear polymer in solution, emulsion, or molten states, or (3) a polymer network, chemically or physically cross-linked. The mineral part can be introduced as (1) a precursor for example tetraethylorthosilicate (TEOS) or (2) preformed nanoparticles. Organic or inorganic polymerization generally becomes necessary if at least one of the starting materials is a precursor. This leads to three general techniques for the preparation of polymer/SiO_2_ nanocomposites according to the starting materials and processing methods: blending, sol-gel processes, and in situ polymerization [27].

**Scheme 1:**
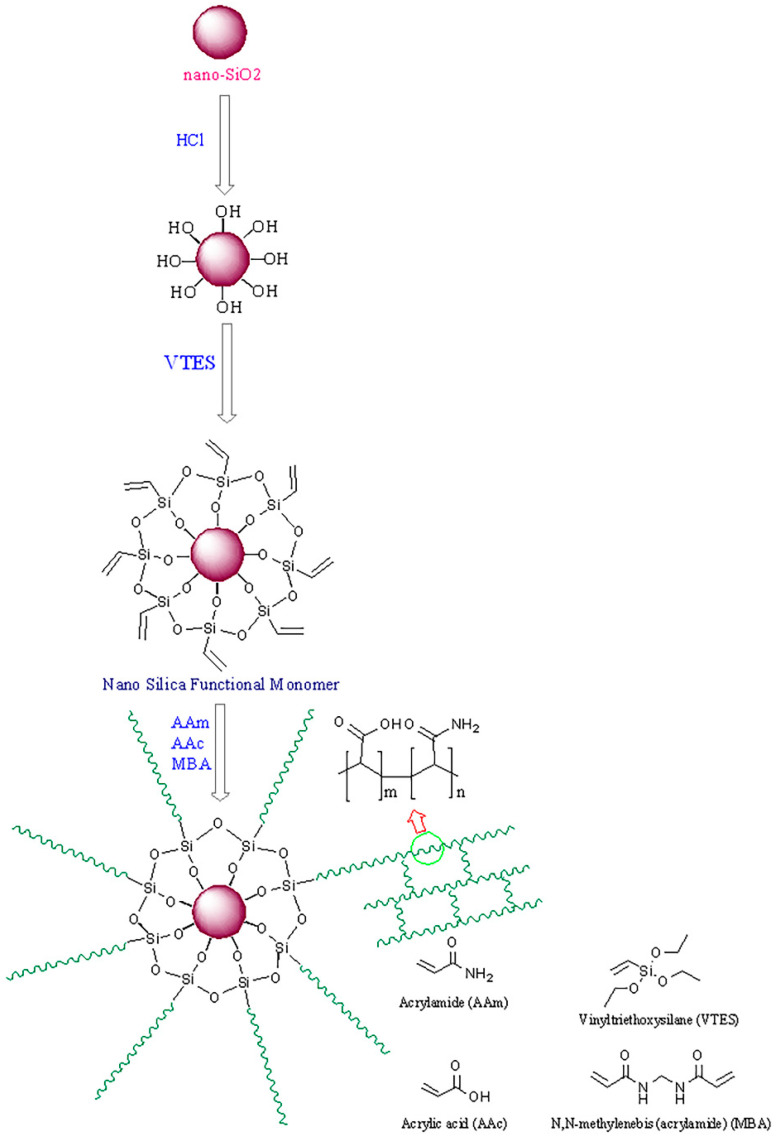
Synthesis of core-shell microgel.

**Scheme 2:**
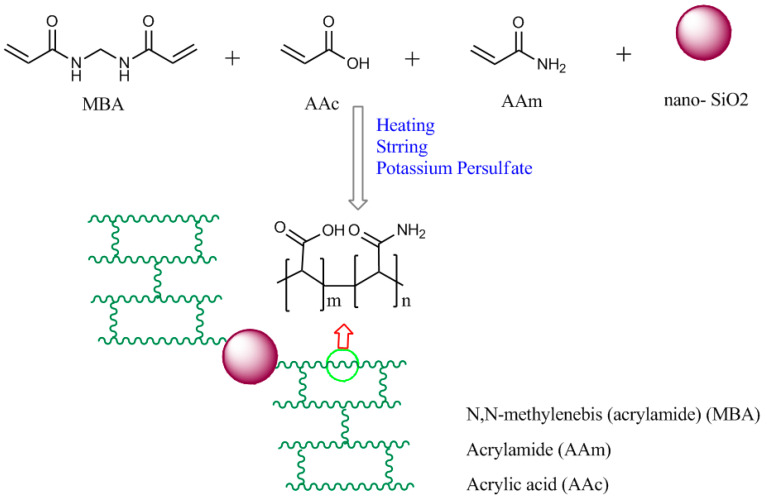
Synthesis of nanocomposite microgel.

**Scheme 3:**
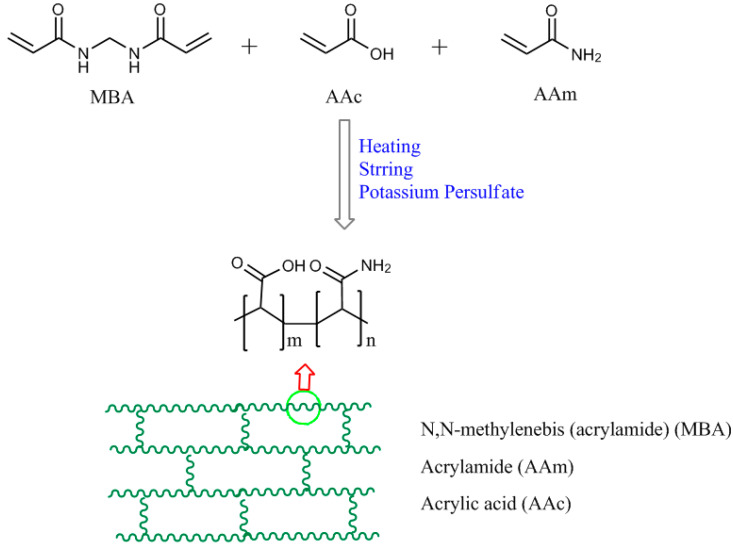
Synthesis of copolymeric hydrogel.

In the following article, we mainly focus on the preparation of microgels containing nano-SiO_2_ with in situ copolymerisation of acrylic acid (AAc) and acrylamide (AAm) as monomers and methylene-bis-acrylamide (MBA) as a crosslinking agent in the presence of nano-SiO_2_ particles. The effect of vinyl functionalization of nano-SiO_2_ with vinyltriethoxysilane on some properties of the prepared microgel samples, such as morphology, thermal stability, glass transition and swelling degree, was also investigated. The grafting mechanism, including the proposed structure of the synthesized samples and a schematic representation of the method for the synthesis of microgel samples and copolymeric hydrogel are shown in Scheme 1, Scheme 2 and Scheme 3. In addition, in this work, synthesis, characterization and swelling studies of copolymeric hydrogel-based on poly (acrylamide) and poly (acrylic acid) have been carried out. The novelty of this research is simple preparation and investigation of surface modification of nano-SiO_2_ on microgel particles’ morphology and their swelling properties. 

## 2. Experimental Section

### 2.1. Materials

Acrylamide (C_3_H_5_NO, AAm, Merck, Darmstadt, Germany, purity: ≥99%), acrylic acid (C_3_H_4_O_2_, AA, Merck, Darmstadt, Germany, purity: ≥99%) and N,N′-methylenebis(acrylamide) (C_7_H_10_N_2_O_2,_ MBA, Sigma-Aldrich, St. Louis, MO, USA, purity: ≥99%), potassium persulfate (K_2_S_2_O_8_, KPS, Panreac Química, Barcelona, Spain, purity: ≥98%), sodium dodecyl sulfate (SDS, Merck, Germany, purity: ≥90%), nanosilica particles (SiO_2_, Us Nano, Houston, TX, USA, purity: ≥99%), hydrochloric acid (HCl, Merck, Darmstadt, Germany, purity = 37%), vinyltriethoxysilane (NaC_12_H_25_SO_4,_VTES, Energy Chemical, Shanghai, China, purity: ≥98%), ammonia (NH_4_OH, Merck, Germany, purity = 25%), ethanol (C_2_H_6_O, Merck, Germany, purity = 99.99%) and distilled water were used in current research. 

### 2.2. Synthesis of Nanosilica Functional Monomer

According to Scheme 1, a 1 g sample of nano-SiO_2_ particles was dispersed in 50 mL of hydrochloric acid, HCl aqueous solution (5%, *v*/*v*) in a 250 mL beaker. The solution was left stirred for 1 h at room temperature. The activated nanoparticles were obtained by centrifugation of solution, washing with distilled water and vacuum drying at room temperature. Then activated nano-SiO_2_ (1 g) was added into the stirred solution of ethanol (60 mL), distilled water (10 mL) and ammonia (1.0 mL) in the round-bottom flask. The mixture was sonicated (24 kHz) for 30 min to better disperse of the nanoparticles. Then, 1.0 mL of vinyltriethoxysilane was added into the stirred solution. The reaction was started at 30 ± 2 °C and stopped after 12 h. The product was nanosilica functional monomer (silane-modified nano-SiO_2_) which was separated by centrifugation and washed with distilled water.

### 2.3. Synthesis of Core—Shell Microgels

The above synthesized nanosilica functional monomer was used as a seed (core) for the synthesis of the AAm/AAc/nanosilica core-shell microgel (Scheme 1). In addition, the nano-SiO_2_ particles were used in the preparation of AAm/AAc/nano-SiO_2_ nanocomposite microgel (Scheme 2). For the synthesis of the AAm/AAc/nanosilica core-shell microgel, firstly, nanosilica functional monomer (500 mg) was dispersed in 100 mL of distilled water by ultrasonic waves (24 kHz) for 30 min. Then, AAm (3.5 g, 49.24 mM), AAc (1.5 g, 20.81 mM), MBA (0.54 g, 3.50 mM ≅ 5% of total monomers) and KPS (198.8 mg, 0.736 mM ≅ 1% of total monomers) as radical polymerization initiator were added to the prepared nanosilica functional monomer aqueous solution and the reaction started at 65 ± 2 °C under stirring conditions and the inert gas, nitrogen (N_2)_ atmosphere. After 4 h the reaction was stopped.

### 2.4. Synthesis of Nanocomposite Microgels

An in situ free radical polymerization technique was used to synthesize AAm/AAc/nano-SiO_2_ nanocomposite microgel. For the synthesis of the nanocomposite microgel sample, 500 mg of nano-SiO_2_ particles (without surface activation and modification) was poured into 100 mL of distilled water and sonicated to better aid the dispersion and then SDS surfactant (25 mg) was added to help the stabilization of the dispersed nano-SiO_2_ particles. After preparation of the nano-SiO_2_ colloidal solution, 3.5 g AAm (49.24 mM) and 1.5 mg AAc (20.81 mM) as comonomer, 0.54 g MBA (3.50 mM ≅ 5% of total monomers) as crosslinking agent, 198.8 mg KPS (0.736 mM ≅ 1% of total monomers) as polymerization initiator were also added to it. The obtained mixture was stirred at 65 ± 2 °C under the inert gas, nitrogen (N_2_) atmosphere. The reaction was complete after 4 h. 

### 2.5. Prepration of Polyacrylamide and Copolymeric Hydrogel

The copolymerization of AAm/AAc in the presence of MBA crosslinker and the absence of nano-SiO_2_ and the nanosilica functional monomer was carried out by using the method and values mentioned in the previous section. To study the water uptake, all synthesized products dried after ethanol washing. Then, they were crushed and sieved to get uniform particle sizes. In addition, the same process was applied to prepare homopolymer hydrogel based on polyacrylamide (PAAm). 

### 2.6. Dynamic Swelling Studies

The pulverized hydrogel, nanocomposite and core-shell microgels in the uniform particle sizes were washed with distilled water and ethanol to remove unreacted starting materials, free oligomers, free polymer and copolymer chains and ungrafted nano-SiO_2_ particles. The dynamic swelling experiment was performed by measuring the water weight of the dried samples. A small amount of superabsorbent samples was taken (0.1 g) and placed in the three beakers. Then, 500 mL of aqueous buffer solution was poured into the beakers. After 5 min the swollen samples were separated by using a filter and then the wet weight was measured carefully. The weight gain as a function of time was taken as the swelling measurement. According to the following Equation (1), the swelling ratio was expressed as the percent weight ratio of the water held in the hydrogel to the dry sample at any instant during swelling.

(1) $$\mathrm{Swelling}\;\mathrm{ratio}\;\left(\%\right)=\frac{W_{t}-W_{d}}{W_{d}}\times 100$$

where W_t_ and W_d_ are the weight of the swollen sample at time t and the weight of the dry sample at time 0, respectively. 

### 2.7. Methods 

After coating the samples with gold film (thickness ≅ 10 nanometers) to obtain a good quality image because the polymers prepared here are not conductive, the morphology of the freeze-dried samples of homopolymer hydrogel, copolymeric hydrogel, nanocomposite and core-shell microgels were examined by a field emission scanning electron microscope (FESEM, Hitachi model S-4160,Daypetronic Company, Tokyo, Japan) at magnifications of 100,000× *g* and 70,000× *g*. Nano-SiO_2_ particles were viewed using a Zeiss Leo 906 (Carl Zeiss Inc., Jena, Germany) transmission electron microscope (TEM) at a magnification of 100,000× *g*. The FTIR spectra of synthesized samples were recorded on Tensor 27 FTIR spectrometers (Bruker Optik GmbH, Ettlingen, Germany) using KBr discs and under strictly constant conditions in the region of 400–4000 cm^−1^. About 5.3 mg of the dry samples were taken and thermogravimetric analysis (TGA)/derivative thermogravimetry (DTG) testing was conducted using an STA409C131F thermogravimetric analyzer (TGA, NETZSCH Company, Germany) in a temperature range of 30 °C–620 °C under the inert gas. In addition, the thermal behavior of samples was determined using a differential scanning calorimeter (DSC-200F3, NETZSCH Company, Germany). Around 4 mg of each sample encapsulated in an aluminum pan. Then the pan was heated from 25 to 200 °C at a heating rate of 10 °C/min under nitrogen purge to measure their glass transition temperature (T_g_) according to ASTM 3418-15 standard.

## 3. Results and Discussion

### 3.1. Morphological Studies

**Figure 1:**
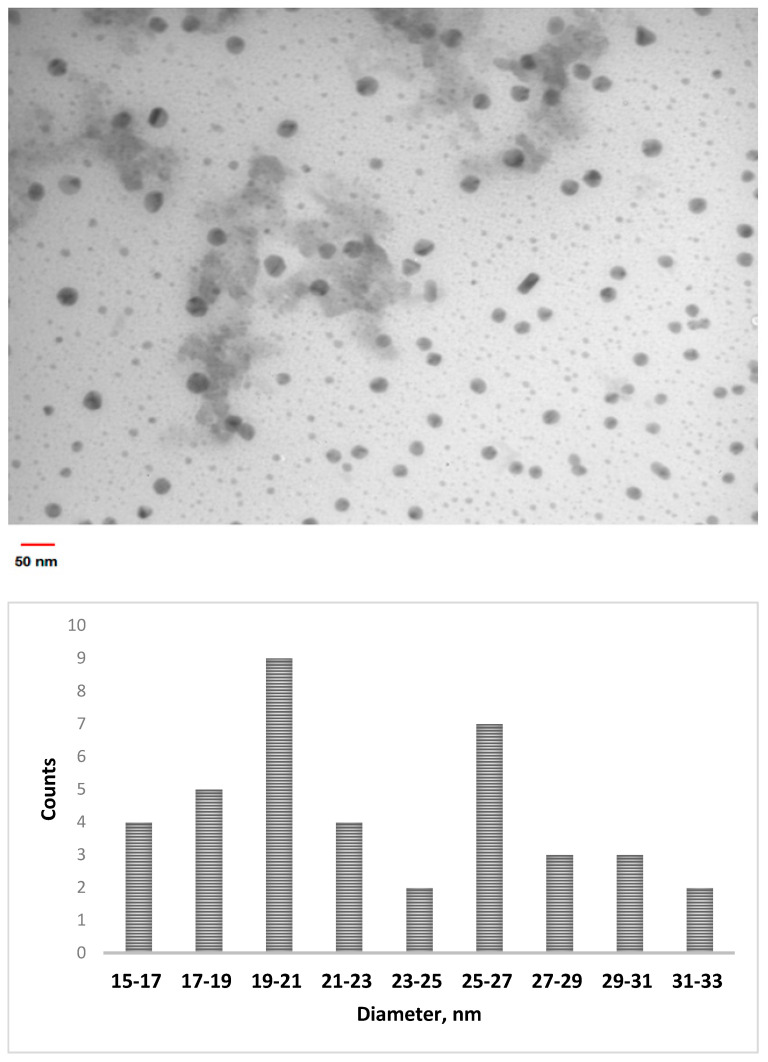
TEM image and bar graphs of diameter ranges of nano-SiO_2._

One of the substantial features of the microgels and hydrogels is their morphologies, which determine their applications and crucial properties such as porosity, water permeation and swelling capacity. TEM results of the nano-SiO_2_ particles are shown in Figure 1. According to TEM image shown in Figure 1, the mean size of the particles was 23 ± 4.6 nm and no particle aggregation was observed.

**Figure 2:**
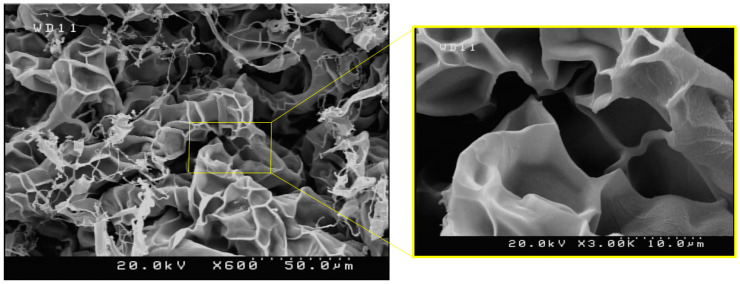
FESEM micrographs of freeze-dried copolymeric hydrogel.

As shown in Figure 2, the copolymerization of AAm and AAc in the presence of MBA crosslinking agent and the absence of nano-SiO_2_ particles resulted in conventional hydrogel formation. The FESEM micrographs of freeze-dried copolymeric hydrogel showed the high porosity and 3D interconnected microstructures like other reported polymeric hydrogel structures. The porosity formation and interconnectivity of the microchannels in the hydrogel structures could be assigned to the crosslinking polymerization in the presence of solvent that dissolves the monomers, but causes precipitation of the formed polymer. Either bulk or solution polymerization can synthesize the hydrogel material. However, bulk polymerization yields a glassy and optically transparent gel with no porosity. In contrast, the solution polymerization produces a hydrogel with porous structures.

**Figure 3:**
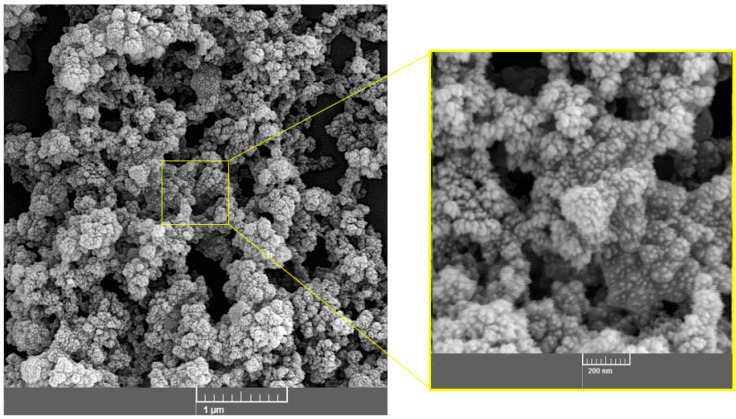
FESEM micrographs of freeze-dried nanocomposite microgel.

As described in the experimental section, when nano-SiO_2_ particles were added to the AAm/AAc copolymerization system, the microstructure and morphology of products were obviously changed from porous hydrogel to nanocomposite microgel with cauliflower-like morphology as shown in Figure 3. FESEM micrographs of the nanocomposite microgel indicated the homogenous dispersion and uniform distribution of the nano-SiO_2_ particles in the AAm/AAc copolymer matrix and most of the nanoparticles are individual and some of them are observable at the surface of the nanocomposite microgel sample. Although the results showed that the AAm/AAc copolymer chains were grafted on the nano-SiO_2_ particles and covalent bonding formed between them. However, the synthesis of AAm/AAc copolymer containing nano-SiO_2_ particles did not result in the formation of complete core-shell morphology. The formation of this special micro-nano structures and the resulting morphology demonstrated that the nano-SiO_2_ particles have also had a co-crosslinking role and have helped three-dimensional structure formation. It is worth mentioning that elimination of MBA crosslinking agent from this copolymerization system, the resulting product did not exhibit any hydrogel properties and a copolymeric solution with very low gel content achieved. 

**Figure 4:**
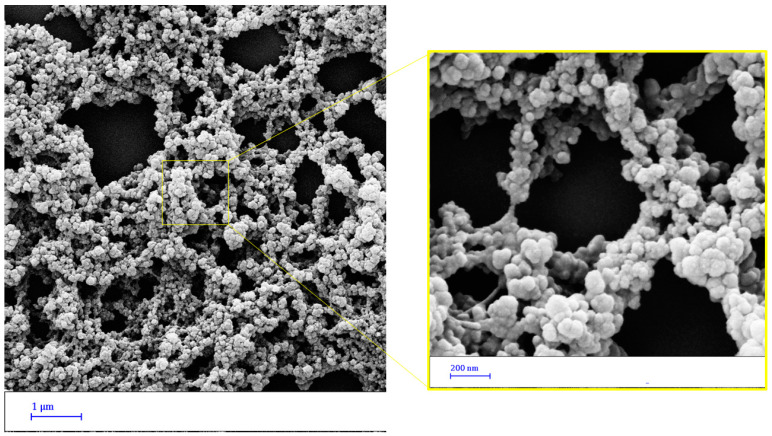
FESEM micrographs of freeze-dried core-shell microgel.

**Figure 5:**
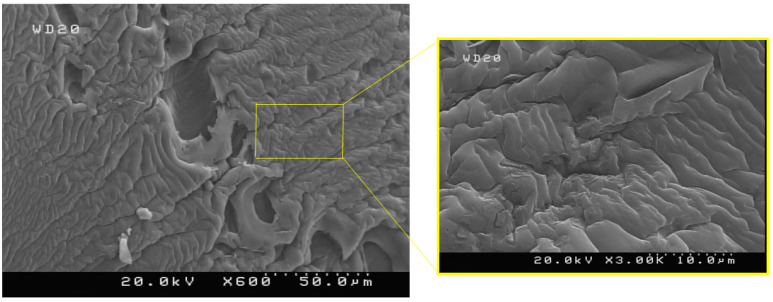
FESEM micrographs of freeze-dried homopolymer hydrogel of PAAm.

The FESEM image of Figure 4 reveals that the copolymerization of AAm and AAc monomores with nanosilica functional monomer or polymerizable nanosilica particle as seed led to core-shell structure. These results demonstrated that a polymerizable vinyl group on nano-SiO_2_ particles not only have worked as a co-crosslinking agent but also as seed have controlled the copolymerization and the product morphology. Core-shell morphology development and shell growth can be summarized as follows. The polymerization of hydrophilic AAm and AAc monomers started from the surface of the vinyl modified nano-SiO_2_ to form an oligomer chain containing shell. During the copolymerization and growth of shell, the propagating copolymer of AAm and AAc anchored on the surface of the nano-SiO_2_ and led to the appearance of core–shell structure. In addition, the aggregation of particles to form core–shell clusters can be attributed to this fact that with growth of a shell layer on the seeds. The adjacent core-shell particles are connected to each other by growing and living chains of AAm/AAc copolymer. Furthermore, as shown in Figure 5, the synthesized homopolymer hydrogel of PAAm did not have any porosity in the FESEM micrographs.

### 3.2. FTIR Analysis

**Figure 6:**
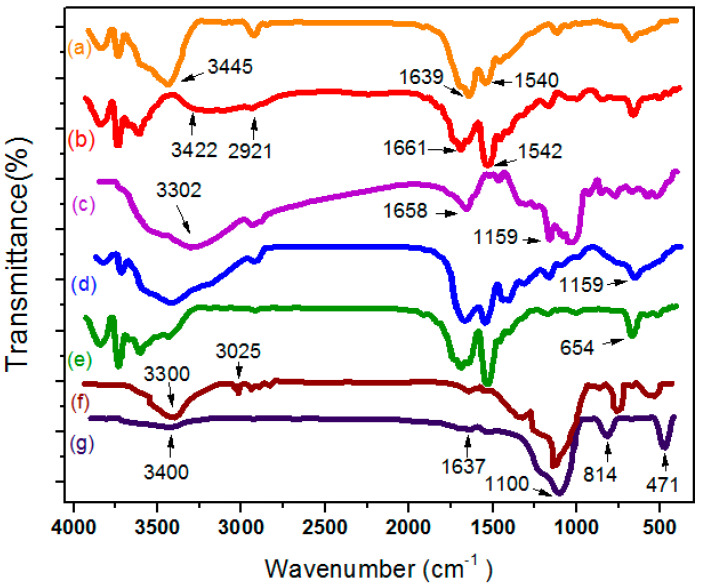
FTIR spectra for: PAAm (**a**), copolymeric hydrogel (**b**), PAAc (**c**), nanocomposite microgel (**d**), core-shell microgel (**e**), nano- SiO_2_ functional monomer (**f**), nano- SiO_2_ particles (**g**).

The characterization of chemical structure of synthesized samples was carried out by FTIR analysis. The spectra of nano-SiO_2_ particles, poly (acrylic acid) (PAAc), PAAm, copolymeric hydrogel of AAm and AAc, nanosilica functional monomer, nanocomposite microgel with cauliflower-like morphology and core-shell microgel were compared and the corresponding results are depicted in Figure 6. The structure of pure PAA was confirmed by absorptions at ν = 3302 cm^−1^ (–OH hydroxyl groups) and ν = 1658 cm^−1^ (–C=O carbonyl groups). The peaks at about 1159 cm^−1^ are attributed to –CO in –COOH of PAA. The characteristic absorption bands of pure PAAm were observed at 3445 cm^−1^ and 1540 cm^−1^ for the N–H and 1639 cm^−1^ for the C=O carbonyl group in the structure of the amide group. In the spectrum of the AAm/AAc copolymer hydrogel, the peak observed at 3422 cm^−1^ corresponds to N-H and O-H stretching. The absorbance at 2921 cm^−1^ is assigned to –C-H stretching of the acrylate group. The peak at 1542 cm^−1^ and at 1661 cm^−1^ are assigned to C=O stretching of the acrylamid groups and acrylate groups, respectively. These absorbance bands in the AAm/AAc copolymer indicated the successful synthesis of copolymeric hydrogel based on PAAm and PAAc. In addition, in Figure 6, the weak bands at 3400 and 1637 cm^−1^ are due to the O–H group on the surface of nano-SiO_2_, and the strong peak observed at 1100 cm^−1^ in the nano-SiO_2_ spectrum is due to the Si-O-Si bonds. Additionally, the bands at 471 cm^−1^ and 814 cm^−1^ in nano-SiO_2_ spectrum represent Si-O bending vibration and stretching vibration, respectively. The strong peak at 3300 to 3400 cm^−1^ and the new peak at 3025 cm^−1^ corresponded to large numbers of Si-O-H group and =CH stretching vibrations in nano-SiO_2_ functional monomer, respectively, which was appeared after the surface modification of nano- SiO_2_ particles. Furthermore, the absorption peak at 654 cm^−1^ is due to the stretching vibrations of Si–C in the structure of microgel with cauliflower-like morphology and core-shell microgel. This suggests that the copolymeric chains of PAAc and PAAm was chemically bonded to the surface of the nano-SiO_2_ particles.

### 3.3. Thermal Stability Analysis

**Figure 7:**
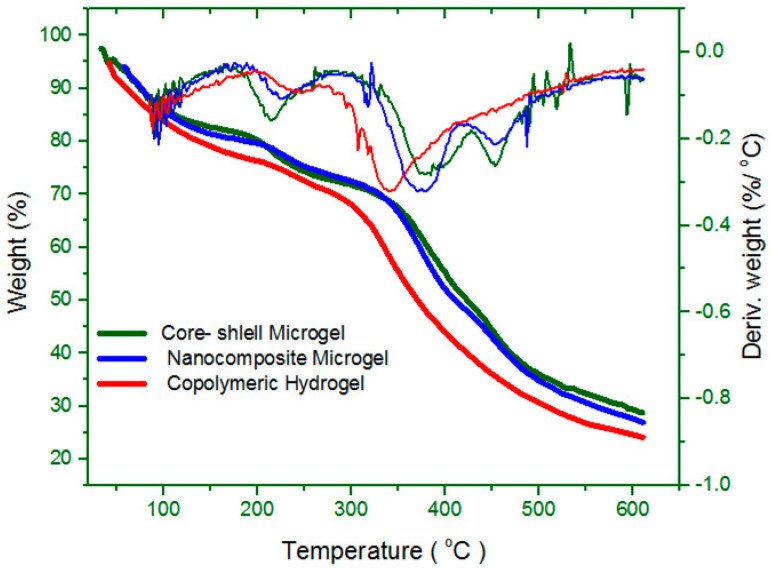
TGA curves of synthesized samples.

Thermal stability of synthesized copolymeric hydrogel and microgel samples were investigated by TGA/DTG at 30–620 °C with N_2_ in the inert atmosphere. Representative TG thermograms of samples along with derivative thermograms (DTG) curve are shown in Figure 7. It is clearly seen from Figure 6 that the weight of the samples continuously decreases as the temperature increases. According to these TGA profiles, three stages of weight loss were observed for both samples of microgels (nanocomposite and core-shell) and two stages of weight loss was seen for copolymeric hydrogel. For both copolymeric hydrogel and microgels, minor weight loss was observed at temperatures less than 240 °C. These weight losses can be attributed to the anhydride formation and the evaporation of volatile solvent or entrapped water in the structure of samples [33]. The second stage of thermal degradation as the main weight loss was observed at 340 °C for the copolymeric hydrogel, 374 °C for the microgel with cauliflower-like morphology and 378 °C for the core-shell microgel. This was assigned to the thermal decomposition of the functional groups in the three copolymeric samples (amide groups in AAm and carboxyl groups in AAc). The final decomposition stage of thermal degradation of the samples at high temperatures can be attributed to the degradation of the C–C bonds in the side and main chain of these three copolymeric samples and destroying of their structures [34]. 

The results showed that the addition of nanosilica particles increased the thermal stability of the microgel samples. At 600 °C, the residual weight percent of copolymeric hydrogels (without the addition of nanosilica particles) was 29.3%. The residual weight percent of the nanocomposite microgel with cauliflower-like morphology was 33.3%. The residual weight percent of core-shell microgel was 31.6%. Briefly, these results indicated that the presence of nanosilica particles resulted in the improvement of the thermal stability of microgel samples compared to pure copolymer. 

**Figure 8:**
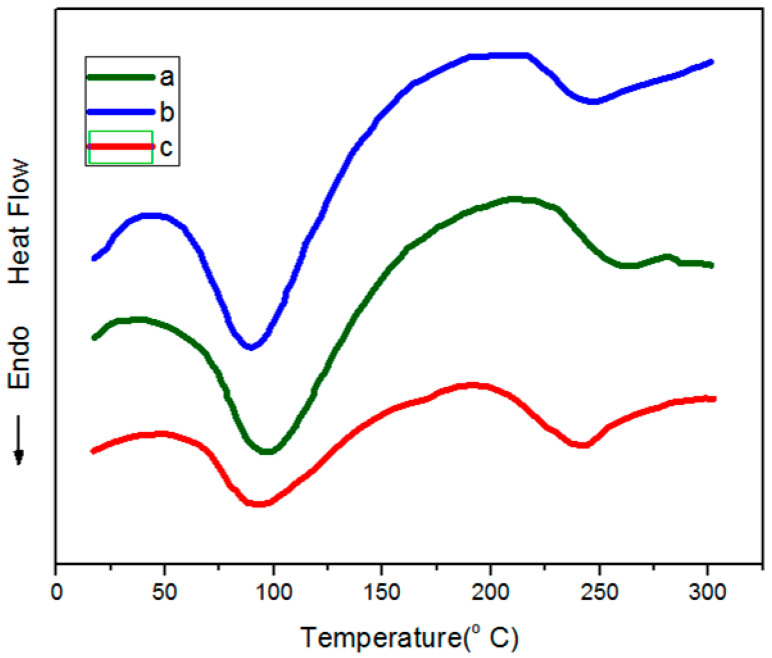
DSC thermograms of the core-shell microgel (**a**), the nanocomposite microgel (**b**) and the copolymeric hydrogel (**c**).

The differential scanning calorimetry (DSC) analysis of the copolymeric hydrogel and microgel samples are shown in Figure 8. The endothermic peaks in the DSC curves of the three samples correspond to their glass transition temperature (T_g_) and volatilization of bound water. For pure copolymeric hydrogel, the glass transition temperature is observed around 88 °C. The T_g_ of nanocomposite microgel with cauliflower-like morphology and core-shell microgel are about 91 and 96, respectively. It is clear that all samples as a random copolymer exhibit only one T_g_. The occurrence of a single peak (T_g_) can be attributed to the miscibility between PAAm and PAAc and the formation intermolecular H-bonding between these polymers. Furthermore, the addition of the nano-SiO_2_ particles shifts the T_g_ of samples slightly to high temperatures resulting in more stable samples. Because there are attractive forces and covalent bonds between the nanosilica and copolymer and the graft and adsorption of copolymer chains on the nanosilica surface decreases their mobility. In addition, the endothermic processes of the samples from 240 °C to 262 °C can be attributed to the volatilization of bound water in the hydrophilic structure of samples.

### 3.4. Swelling Studies

**Figure 9:**
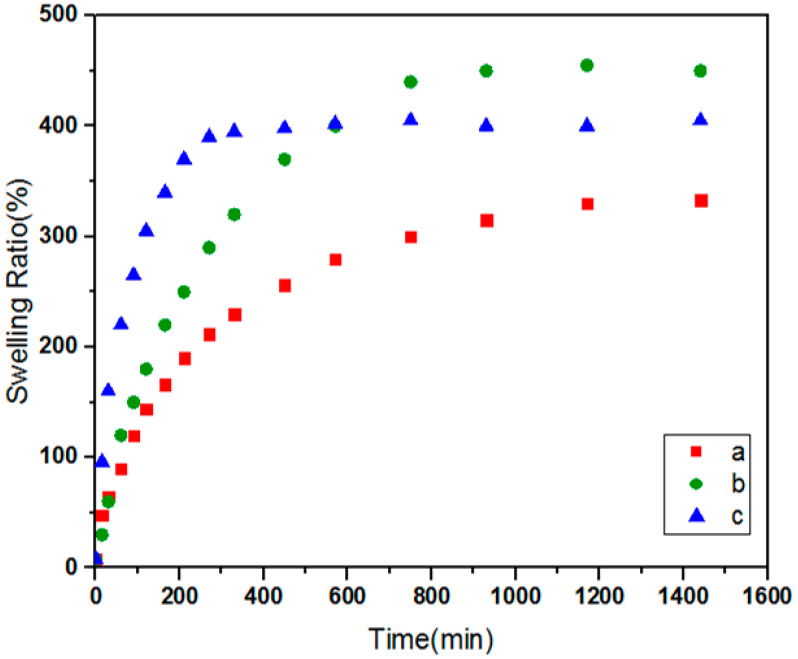
Swelling behavior of the copolymeric hydrogel (**a**), the core-shell microgel (**b**), and the nanocomposite microgel (**c**).

In order to study the swelling behavior, samples were allowed to swell to equilibrium in buffer solution of pH 7.4 at a room temperature and the swelling kinetics of these samples were investigated. The dynamic swelling behavior of the samples are shown in Figure 9. As can be seen from their swelling behavior in this figure, the swelling of samples increases with time until a certain point when it becomes constant. These constant values are taken as the equilibrium swelling and are 333% for the copolymeric hydrogel, 405% for the microgel with cauliflower-like morphology and 430% for the core-shell microgel. The ability of water absorbency of the samples prepared in this study arises from the two hydrophilic functional groups, -COOH of the AAc and -CONH_2_ of the AAm units attached to the copolymeric backbone of these samples. While the resistance of these samples with many hydrophilic functional groups to dissolution arises from the presence of the MBA crosslinker and three-dimensional network structure. It is well known that PAAm is nonionic and insensitive to pH of the medium but PAAc is a pH- sensitive polymer. All our samples formed by the homogenous copolymer of AAc and AAm with MBA as a crosslinker where the carboxylic acidic groups of AA which bound to the copolymer chains made the samples pH sensitive. Thus, at a pH lower than the dissociation constant, pK_a_ of PAAc (about 4.3) the degree of ionization of the carboxylic acid group is small and most of them are in –COOH form which can form a hydrogen bond with -COONH_2_ side groups of AAm units leading to shrinking of hydrogel or microgel samples. In contrast, at neutral or basic pH (or pH greater than 4.3) such as a pH value of 7.4 in this study, AAc units in backbone of prepared copolymeric samples are negatively charged due to the ionization and the deprotonation of COOH groups. Thus, all AAc/AAm copolymeric samples resulted in expansion of networks and swell to a great degree at this pH condition (pH = 7.4) because of the electrostatic repulsion among the carboxylate anions (–COO^–^). Meanwhile, as shown in Figure 2, the copolymeric hydrogels show a porous network structure in character that makes it easier for water to diffuse in or out of the gel matrix. The porosity that itself has a great influence on swelling behavior can be generated by electrostatic repulsive forces among the similarly charged carboxyl groups along copolymeric segments during the copolymerization process. Whereas as shown in Figure 5 there are no porosity in the FESEM micrographs of homopolymer hydrogel of PAAm and it has a relatively dense structure. In addition, from the swelling plots in Figure 8, it is clear that the water absorbency of the microgel samples is much more than the copolymeric hydrogel. 

According to the morphology study and the FESEM results in Figure 2, Figure 3 and Figure 4, particulate character of microgels and the existence of long distance among aggregations of particles lead to rapid swelling and the increase of porosity and therefore increasing the degree of swelling. The differences in the kinetics of swelling for the core-shell microgel and the nanocomposite microgel can be attributed to this fact that according to Scheme 1 and Scheme 2 nanosilica functional monomer as seed have not been played as crosslinking agent between chains and particles but nano-SiO_2_ particles in the nanocomposite microgel have had the crosslinking role. As a result, the swelling rate of the core-shell microgel was similar to the copolymeric hydrogel due to a similar degree of crosslinking and chemical similarity of the shell and copolymeric sample. The high swelling rate and low swelling ratio of nanocomposite microgel in comparison with the core-shell microgel could be because of the smaller particles and high degree of crosslinking, respectively. 

## 4. Conclusions

Synthesis, characterization, and morphological, structural and swelling studies of novel microgels containing nano-SiO_2_ and copolymeric hydrogel based on PAAm and PAAc was successfully conducted. The FESEM micrograph of freeze-dried copolymeric hydrogel show the high porosity and 3D interconnected microstructures. The interconnectivity of the microchannels in the hydrogel structures could be assigned to the MBA crosslinking of poly (AAm-co-AAc) chains. It was found that introducing only a small amount of nanosilica into the copolymerization system can change the morphology of products from porous hydrogel (hydrogel particle). The presence of silanol and siloxane groups on the nano-SiO_2_ surface led to better control of morphology of the microgel and formation of complete core-shell due to hydrophilicity, compatibility and because of polymerizable vinyl group of VTES on nanosilica particles. TGA revealed that the presence of nanosilica particles in nanocomposite microgel with cauliflower-like morphology and core-shell microgel resulted in the improvement of the thermal stability compared to copolymeric hydrogel. The glass transition temperature (T_g_) for pure copolymeric hydrogel, nanocomposite microgel and core-shell microgel were observed by DSC around 88, 91 and 96 °C, respectively. In addition, water absorbency of the microgel samples was much more than copolymeric hydrogel due to their particulate character. High swelling rate and low swelling ratio of nanocomposite microgel in comparison with the core-shell microgel could be because of the smaller particles and high degree of crosslinking, respectively.

## Data Availability

The data presented in this article are available within the article.
